# Cocoa plantations are associated with deforestation in Côte d’Ivoire and Ghana

**DOI:** 10.1038/s43016-023-00751-8

**Published:** 2023-05-22

**Authors:** Nikolai Kalischek, Nico Lang, Cécile Renier, Rodrigo Caye Daudt, Thomas Addoah, William Thompson, Wilma J. Blaser-Hart, Rachael Garrett, Konrad Schindler, Jan D. Wegner

**Affiliations:** 1 EcoVision Lab, Photogrammetry and Remote Sensing, ETH Zurich, Zurich, Switzerland; 2 Department of Computer Science, University of Copenhagen, Copenhagen, Denmark; 3 Earth and Life Institute, UCLouvain, Louvain-la-Neuve, Belgium; 4 Department of Geography and Conservation Research Institute, University of Cambridge, Cambridge, UK; 5 Nature-Based Solutions Initiative, Department of Biology, University of Oxford, Oxford, UK; 6 School of Biological Sciences, University of Queensland, St Lucia, Brisbane, Queensland Australia; 7 Data Science for Sciences, Institute for Computational Science, University of Zurich, Zurich, Switzerland

**Keywords:** Forestry, Agroecology, Biodiversity

## Abstract

Côte d’Ivoire and Ghana, the world’s largest producers of cocoa, account for two thirds of the global cocoa production. In both countries, cocoa is the primary perennial crop, providing income to almost two million farmers. Yet precise maps of the area planted with cocoa are missing, hindering accurate quantification of expansion in protected areas, production and yields and limiting information available for improved sustainability governance. Here we combine cocoa plantation data with publicly available satellite imagery in a deep learning framework and create high-resolution maps of cocoa plantations for both countries, validated in situ. Our results suggest that cocoa cultivation is an underlying driver of over 37% of forest loss in protected areas in Côte d’Ivoire and over 13% in Ghana, and that official reports substantially underestimate the planted area (up to 40% in Ghana). These maps serve as a crucial building block to advance our understanding of conservation and economic development in cocoa-producing regions.

## Abstract (web summary)

The exact location and extent of cocoa plantations in Côte d’Ivoire and Ghana, the world’s largest producers, remain unknown in spite of their social, economic and environmental relevance. New satellite-based high-resolution maps generated through a deep learning framework link cocoa cultivation with deforestation in protected areas and show that official reports underestimate the total planted area.

## Main

Cocoa is grown by an estimated two million farmers in West Africa, who supply a complex network of middlemen, including private and public companies, which renders the supply chain rather opaque^1–3^. With an average farm size of three to five hectares^4,5^ and an estimated income of less than one dollar per day, nearly all cocoa farmers live under the poverty line^6^. In this context, deforestation in West African Upper Guinean forests (a biodiversity hotspot^7^) has occurred in waves across the twentieth and twenty-first centuries^8^. Cocoa-driven deforestation has played a substantial role in this and has been catalysed by migration from savannah regions, land availability and tenure constraints for residents of existing cocoa production areas, and the higher productive potential of recently cleared land^1,9^.

In recent years, corporate sustainability efforts have been initiated to reduce cocoa-driven deforestation and improve cocoa yields, including by promoting agroforestry^10^. While cocoa certification programmes have improved farm productivity and income, there is no conclusive evidence of their impact on agroforestry or deforestation. Furthermore, more ambitious supply chain targets to eliminate sourcing of deforestation-linked cocoa face major implementation challenges due to difficulty in monitoring and tracking cocoa expansion into forests^11,12^. Cocoa has been the primary driver of deforestation in these countries, alongside mining, selective logging and other crops^2,9,13^. However, the extent to which cocoa has directly and indirectly replaced forest has been uncertain.

Current map products either are derived from small reference datasets and are of low precision, or rely on manual georeferencing and are costly to update^12,14^. The production of accurate, high-resolution maps of cocoa-growing areas is currently missing. Up-to-date maps could greatly enhance efforts to halt deforestation by highlighting high-deforestation-risk sourcing areas for cocoa, verifying production quantities and estimating on-farm versus off-reserve production area. Beyond deforestation, the spatial extent of cocoa production could be linked with more readily available data on production quantities to inform more targeted extension activities.

Here we present a large-scale, high-resolution cocoa growing map spanning Côte d’Ivoire and Ghana, generated by satellite image analysis with a deep neural network. Deep learning has matured and surpassed traditional hand-crafted feature detectors in countless remote sensing tasks including vegetation height mapping^15^, localizing fires^16^, predicting photovoltaic solar facilities^17^ and crop identification^18,19^. When trained on large reference corpora, deep models offer an unprecedented ability to recognize visual patterns in unseen data.

**Fig. 1:**
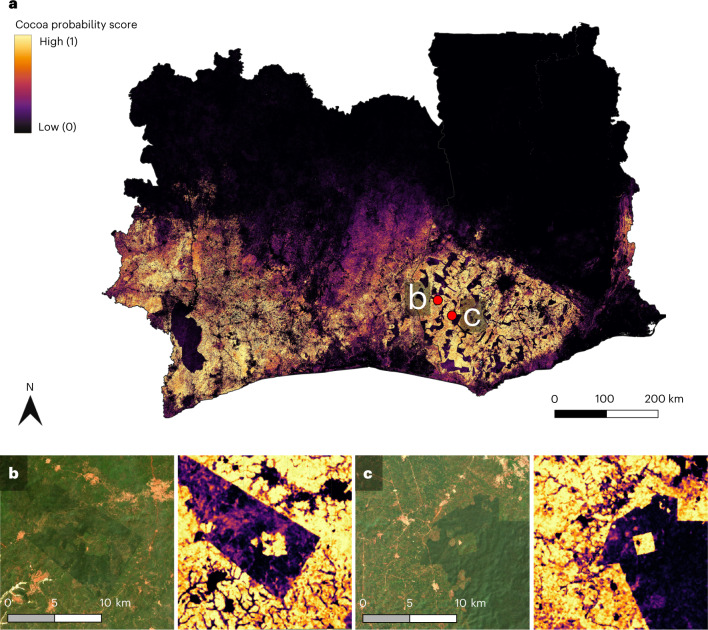
Cocoa map for Côte d’Ivoire and Ghana. **a**, Probability map with 10 m × 10 m ground sampling distance. The map indicates detection confidence in a range [0, 1]—that is, values near 1 indicate that model predictions across most time steps agree on the presence of cocoa, and values near 0 indicate that they agree on the absence of cocoa. **b**,**c**, Two forested regions in Ghana where our model has detected cocoa farming (satellite images and confidence maps). The locations of these forest regions are shown with red dots in **a**. Copernicus Sentinel-2 data modified from ref. ^59^.

For this work, we have trained a neural network on a dataset of >100,000 georeferenced cocoa farms to map cocoa plantations at the country scale. We leveraged publicly available optical satellite imagery as input in a twofold way. First, we trained a neural network to predict canopy height in the sub-Saharan region using ground truth acquired from the GEDI mission^20^. Second, we trained a deep neural network on the same satellite imagery and a large corpus of polygons delineating cocoa farms, using the canopy height map as an additional input for the network, thus introducing an explicit prior on the plant height. With the help of a team in Côte d’Ivoire, we validated our map in situ in a three-month-long campaign, accompanied by further verification with a partially hand-labelled test set in Ghana. Instead of a single binary cocoa map, we created a probability map by aggregating predictions from a model ensemble and from repeated observations of the same location, as model ensembles have been shown to yield more reliable uncertainty calibration and often also improved predictive skill^21^. The final map, alongside two examples of cocoa grown in different protected areas, is depicted in Fig. 1.

We illustrate the utility of our map by analysing planted area as well as farming practices and sustainability efforts for reducing deforestation, highlighting the need for land cover mapping independent of farmers, industry and governments. We also identify regional areas that are exposed to poor growing conditions.

## Results

### Evaluation

We first demonstrate the reliability of our ensemble model with four standard accuracy metrics. Precision (user’s accuracy) measures the proportion of correctly classified pixels among all pixels assigned to a class. Recall (producer’s accuracy) is the proportion of correctly classified pixels among all pixels that truly belong to a class. We additionally report accuracy (the overall fraction of correctly classified pixels) and *F*_1_ score (defined as the harmonic mean of precision and recall for a class *c*) as summary statistics:

1 $${F}_{1}^{c}=2\times \frac{{{{\rm{recall}}}}\times {{{\rm{precision}}}}}{{{{\rm{recall}}}}+{{{\rm{precision}}}}}.$$

**Fig. 2:**
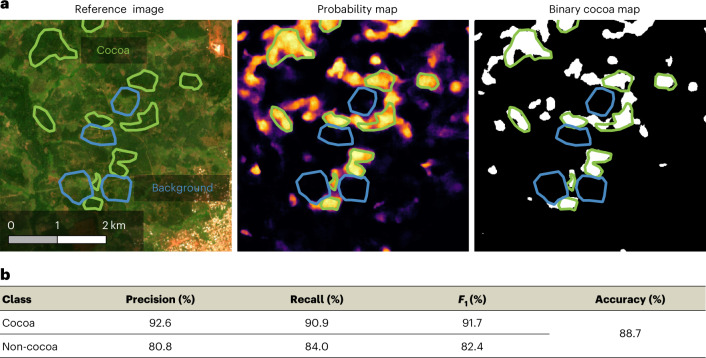
In situ evaluation. **a**, Sites from our in situ test set around Divo, Côte d’Ivoire. Left to right, a satellite image with the reference data, confidence prediction map and binary cocoa map at a confidence threshold of 0.65. The green sites are mapped cocoa farms, and the blue sites are verified non-cocoa sites. In the binary map, each value is either 0 or 1 (that is, each area is classified as either non-cocoa (0) or cocoa (1)), whereas in the probability map the values can be in between. **b**, Quantitative performance of cocoa detection (confidence threshold, 0.65). Copernicus Sentinel-2 data modified from ref. ^59^.

To avoid evaluation bias ^22^, we present all metrics for both classes (cocoa and background) in Fig. 2b and showcase one example of our in situ test set in Fig. 2a.

A confidence map brings several benefits. One notable advantage is that one can calibrate the optimal threshold for binary cocoa presence/absence mapping with an additional, much smaller validation set for specific regions or applications. We do this to evaluate our cocoa map against two independent, binary validation datasets. In Côte d’Ivoire, we manually labelled over 2,000 polygons and verified these estimates with on-farm visits. Additional information on this in situ dataset is given in the Methods. For Ghana, we acquired georeferenced cocoa polygons from an independent commercial data provider. While our in situ test set covers various regions in both countries, it is restricted to the areas around major cities in Côte d’Ivoire, since it was infeasible to collect a statistically rigorous (stratified) random sample^23^ over a region of this scale.

Compared with previous mapping efforts, our approach offers several advantages. First, by using an end-to-end trainable framework, feature selection is automated. Our approach boosts all metrics by large margins compared with the only other large-scale cocoa map we are aware of^14^, improving precision and recall by more than 30% and 8%, respectively. In terms of mapping effort, existing accountability maps^24,25^ rely on extensive collaborations with cocoa cooperatives to create, update and maintain databases of cocoa farms, which impedes their extension to underrepresented regions, both within countries and beyond. In contrast, our mapping system is naturally expandable to areas that have not previously been mapped. Our mapping system can be adjusted with a small amount of local reference data when expanded to a new area that contains similar plant characteristics. For example, our map detects cocoa plantations in regions that have so far been ignored in official figures^26^ (such as the Volta region in Ghana).

### Planted area

With our cocoa map, we were able to calculate the total planted area in Côte d’Ivoire and in Ghana and compare it to official figures. We computed the best threshold at 0.65 according to a held-out validation set—that is, all values above 65% in the probability map are classified as cocoa areas and all others as non-cocoa areas. Importantly, the probabilistic mapping approach makes it possible to select the threshold that maximizes the F_1_ score over the validation set, thus balancing the expected precision and recall on unseen data. We empirically found maximizing the F_1_ score to be more robust than direct matching of false negative and false positive counts: under practical conditions, where the training and test data are not perfect random samples from the underlying distribution, it leads to a lower area bias. The corresponding curve, where the F_1_ score is plotted against different thresholds, can be found in the Supplementary Information.

To estimate planting areas, the uncertainty quantification via our model ensemble approach has several advantages. It has been argued that plain pixel counting in a binary classification map can be problematic for area estimation^23^. However, our final map consists of continuous probability values, similar to model-based area estimation^27^, that can be thresholded in a post-processing step to minimize the bias of the area estimates. The theoretical scale factor between the true and estimated areas is $\frac{{{{\rm{precision}}}}}{{{{\rm{recall}}}}}$. For our test results, that factor amounts to 1.02—that is, a difference of less than 2%. Moreover, each ensemble member produces its own (continuous) cocoa map and can be thresholded separately, thus drawing multiple samples from the distribution of area estimates. Contrary to naive pixel counting, we can thus characterize the uncertainty of the planted area estimate by computing a mean area with an associated standard deviation and confidence interval (CI), assuming an underlying *t*-distribution. The alternative—computing area estimates via optimally selected, stratified samples^23,28^—is difficult for large-scale mapping efforts, particularly when the map resolution is high (in our case 10 m). To obtain such samples, either one would need access to images of even higher resolution anywhere in the region of interest to perform photo interpretation, or one must collect in situ data across entire countries, which is often not possible due to the difficulties of accessing randomly sampled locations that may lie in difficult terrain, lack infrastructure or be subject to land rights.

In 2021, the mean total area under cultivation was 4.45 Mha (95% CI, 3.95–4.95 Mha) in Côte d’Ivoire and 2.71 Mha (95% CI, 2.21–2.89 Mha) in Ghana, corresponding to 13.8% of Côte d’Ivoire’s land area and 11.4% of Ghana’s. The detected cocoa plantings align well with climatically suitable growing regions in both countries^29^, although we have not restricted the detection to those areas a priori, as in previous mapping projects^14^. Compared with the official FAOSTAT figures^30^, our result deviates only marginally from the harvested area (average 2017–2020) in Côte d’Ivoire but differs drastically for Ghana’s total harvested area. FAOSTAT reports 4.47 Mha of harvested area in Côte d’Ivoire (that is, 0.5% more than our estimate) and only 1.63 Mha in Ghana (39.8% less than our estimate).

**Fig. 3:**
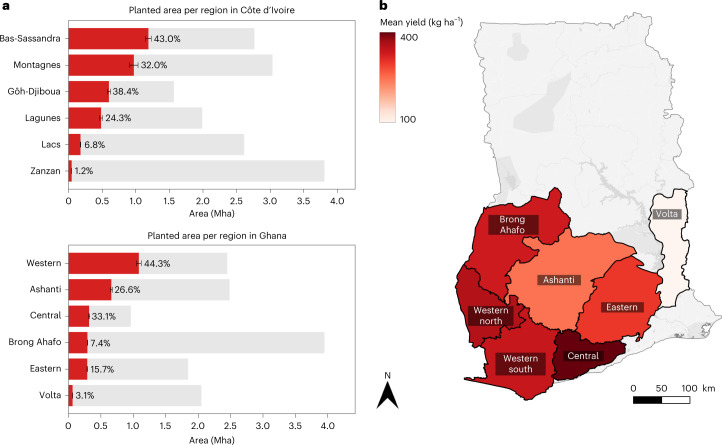
Planted area, mean annual yield and correlation between growing area and production volume per region. **a**, Comparison of planted area and total area per region in Côte d’Ivoire (top) and Ghana (bottom). The grey bars represent the total area of each region, while the red bars show the mean absolute cocoa-planted area for the ten models (the black error bars show the corresponding standard deviations). The percentages indicate the relative amount of cocoa-planted area to the total area per region. **b**, Regional yield differences measured in kilograms per hectare in Ghana. For the Volta region, we obtained production data for only a single subdivision, hence the low average yield.

While the country-wide harvesting numbers are impressive by themselves for a single agricultural commodity, zooming in on a regional level further reveals the massive impact of cocoa cultivation on the two countries. As shown in Fig. 3a, the highest proportions for a single region are 43.0% in Côte d’Ivoire (Bas-Sassandra; 95% CI, 1.16–1.22 Mha) and 44.6% in Ghana (Western; 95% CI, 1.06–1.11 Mha), leaving little to no forested area in agricultural regions. This reveals the extent to which cocoa production has replaced native forests, which is associated with biodiversity loss, local and global climate impacts, and the loss of multiple resources supporting food security and livelihoods.

### Production

We used production data obtained from the Ghana Cocoa Board^31^ and averaged over the years 2017 to 2020 to compare planted area with production data on a regional level in Ghana (production data at the subnational level was not available for Côte d’Ivoire). By dividing production by planted area in every region, we obtained local mean yield estimates, important for farming practices, sustainability and regeneration efforts. Mean annual yields ranged from 250 kg ha^−1^ in the Ashanti region to over 380 kg ha^−1^ in the Central region. The mean yield for the Volta region (120 kg ha^−1^) needed to be treated with care in our analysis, because production is reported for only one of the four cocoa-growing districts. In Fig. 3b, we highlight differences in farming productivity among the cocoa-growing regions in Ghana. As an example, even though the Western region contributes >40% of the total growing area within the Ghana Cocoa Board boundaries, the average yield is lower than that of the Central region, suggesting the potential to improve farming practices. Even more extreme is the Ashanti region, where the annual yield is as low as 250 kg ha^−1^. On average, we obtained a mean annual yield of 320 kg ha^−1^ for Ghana. Earlier studies have reported average cocoa yields in the range of 400 to 530 kg ha^−1^ (refs. ^5,32–34^), which is notably higher than our estimate. These earlier numbers are based on small sets of field samples, which may be biased towards farms with above-average productivity^5^. Our yield estimates may also be slightly lower due to young planted farms detected by our map that may not be productive yet, therefore bringing down the average yield per unit area.

### Protected areas

Côte d’Ivoire and Ghana continue to experience high forest loss. Côte d’Ivoire is estimated to have lost more than 90% of its forest cover since 1950, while Ghana has incurred forest losses of more than 65%^12^. Forest clearance rates reached a high in 2018, increasing by 60% over 2017 in Ghana and by 26% in Côte d’Ivoire, the two highest increases in annual deforestation rates worldwide^35^. However, deforestation rates have fluctuated since 2018.

Here we examine to what extent cocoa replaced native forest. Our map enables an accurate assessment of cocoa-related deforestation within protected areas mapped in the World Database on Protected Areas (WDPA)^36^. The WDPA includes various types of protected areas, including strict nature reserves, national parks and protected landscapes. Across all management categories, there are 242 protected areas in Côte d’Ivoire and 286 in Ghana. We found a total cocoa-planted area of >1.5 Mha located in protected areas: 1.3 Mha in Côte d’Ivoire (30% of the total cocoa area of the country) and 0.2 Mha in Ghana (7% of the total cocoa area). These numbers correspond to >13.6% of the overall protected area in Côte d’Ivoire (9.8 Mha) and >4.5% in Ghana (3.7 Mha). Using annually updated data on forest cover loss^37^, we can directly relate forest loss of over 360,000 ha in protected areas (including classified forests) to cocoa cultivation in Côte d’Ivoire from 2000 to 2020. Given an overall forest loss of 962,000 ha since 2000, cocoa is directly or indirectly responsible for almost 37.4% of forest loss in protected areas. Similarly, we can trace back 26,000 ha of cocoa-driven deforestation in protected areas in Ghana, corresponding to 13.5% of the total forest lost in protected areas (193,000 ha) since 2000.

**Fig. 4:**
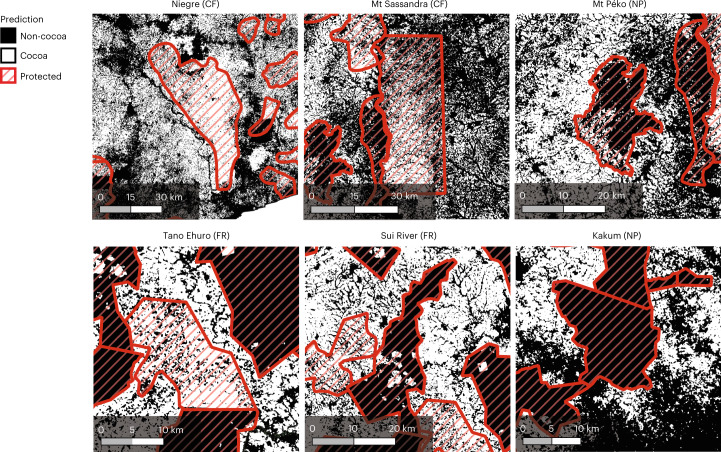
Cocoa encroachment into protected areas. Maps of selected protected areas.

We further broke down the numbers to individual protected areas. Table 1 lists the total cocoa area and the corresponding relative land cover and deforestation percentage for five selected areas per country. Figure 4 visually shows the encroachment. The results reveal that for certain protected areas in the WDPA, up to 80% of the surface is covered by cocoa plantations. They also show a large difference in deforestation across protected area types that requires considerably more investigation.

**Table 1:** Selected protected areas

Côte d’Ivoire				Ghana			
Protected area	Cocoa (ha)	Land cover (%)	Deforestation (%)	Protected area	Cocoa (ha)	Land cover (%)	Deforestation (%)
Niegre (CF)	108,256	81.8	86.6	Tano Ehuro (FR)	16,275	77.6	82.2
Scio (CF)	90,418	68.2	78.3	Manzan (FR)	15,512	56.1	48.6
Mt. Sassandra (CF)	54,946	49.0	55.8	Upper Wassaw (FR)	3,198	23.6	14.6
Mt. Péko (NP)	6,479	21.5	19.3	Sui River (FR)	3,497	9.84	39.3
Marahoué (NP)	2,789	18.0	13.2	Kakum (NP)	256	1.0	21.0

Land cover is the proportion of cocoa within the protected area. Deforestation indicates the proportion of cocoa grown on deforested areas. CF, classified forest; FR, forest reserve; NP, national park. Forest reserves are categorized as protected areas with sustainable use of natural resources. The full list can be found in Supplementary Table 2.

The most deforested protected areas in Côte d’Ivoire are the classified forests of Niegre, Scio and Mt Sassandra, with 81.8%, 68.2% and 49.0% of their area under cocoa cultivation, respectively. All three of them have been exposed to illegal farming for decades^13,38^. Similarly, forest reserves such as Tano Ehuro, Manzan and Upper Wassaw in Ghana have severe forest clearing^39,40^, with cocoa expansion occurring on 23% to 77% of their surface. These high levels of deforestation in protected areas confirm and extend what has been found in Abu et al.^14^ for a very small subset of protected areas. For protected areas “of highest protection” in Ghana (for example, Kakum National Park), our map detects almost no illegal cocoa plantations (1.0%). However, some of the national parks in Côte d’Ivoire are highly affected by illegal cocoa farming. In line with recent literature^6,41,42^, we were able to quantify the spatial extent of cocoa plantations within the protected areas, such as over 6,400 ha in Mt Péko National Park and over 2,700 ha in Marahoué National Park. Yet, Tai National Park, a World Heritage Site and one of the largest protected areas in Côte d’Ivoire, has experienced very little deforestation for cocoa.

Part of the reason for the high overall deforestation rate—aside from the many underlying drivers outlined elsewhere—is that some of the protected areas in the WDPA had already been degazetted early in the study period, thereby allowing cocoa production. In other regions (particularly in Ghana), some villages and farms known as “admitted communities and/or farms" are legally allowed to remain in the forest reserves and to farm within delineated boundaries. However, it is known that these rights have been misused to further expand into remaining forests^43^.

### Vegetation health

**Fig. 5:**
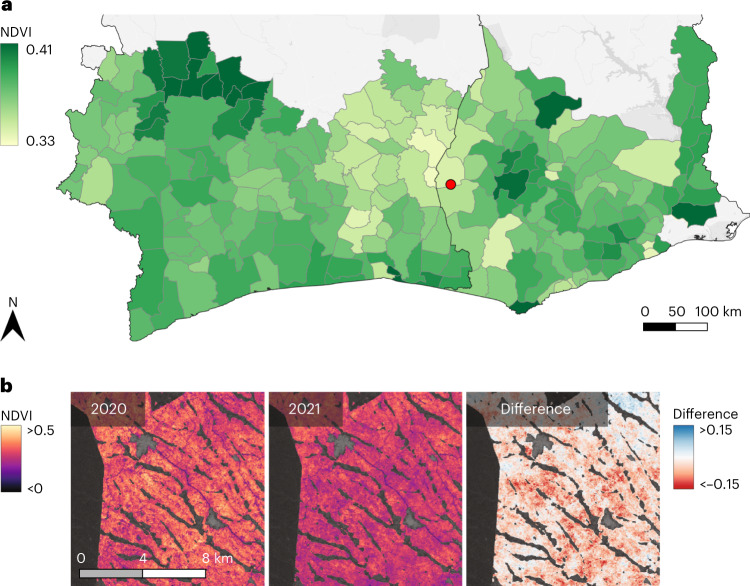
Vegetation health of cocoa, measured by NDVI. **a**,**b**, Computing NDVI per district using only locations with actual cocoa plantings diminishes biases due to other types of vegetation (**a**) and allows targeted actions up to the farm level when comparing at the native map resolution (**b**). The red dot in **a** indicates the approximate location in **b**.

Our map makes it possible to compute further vegetation parameters specifically for regions where cocoa is grown, while excluding other vegetation—that is, plants that are not mapped as cocoa by our model. Naturally, the computed values can be influenced by shade trees and other vegetation within agroforestry systems. This segregation of cocoa and other vegetation allows us to use the normalized difference vegetation index (NDVI) to monitor cocoa health on a large scale, with analyses at either the pixel or district level, and to find regions where resources could be best used to improve the conditions of cocoa plantations. To demonstrate this, we measured vegetation health in terms of the NDVI. This index is based directly on the absorption of photosynthetically active radiation by leaves and the re-emission of near-infrared radiation with too low photon energy. The index is defined as

2 $${{{\rm{NDVI}}}}=\frac{{{{\rm{NIR}}}}-{{{\rm{RED}}}}}{{{{\rm{NIR}}}}+{{{\rm{RED}}}}},$$

where NIR and RED are the spectral reflectances in the near-infrared and red spectral bands, respectively. Higher NDVI at similar leaf areas corresponds to better health. We showcase a fine-grained analysis at the district level for Côte d’Ivoire and Ghana, with the potential to identify local farming practices that promote the health of cocoa plants. In Fig. 5a, we depict the average NDVI per district from October 2018 to December 2021. The values range from slightly below 0.34 to slightly above 0.41, with an average of 0.38. There are clear regional differences. In particular, the border region between the northeast of Côte d’Ivoire and the west of Ghana exhibits a cluster of lower NDVI values. We note that in Ghana the Western region is the principal cocoa-growing area, with over one million hectares of cocoa. Yet, the low NDVI suggests worse plant health and lower productivity than, for instance, the Ashanti and Central regions. This could be further investigated, as the NDVI values could also be influenced by shade trees within agroforestry systems or by local climatic characteristics. While the NDVI at the district level is appealing to quickly identify larger areas that may have to be further investigated, it simultaneously becomes less expressive due to averaging effects over large areas. However, our map allows for fine-grained analysis up to the farm or even subhectare level. As seen in Fig. 5b, we can compute vegetation health indices per pixel and year as an average over three months to reduce measurement noise in the corresponding Sentinel-2 bands. These maps can be used individually to compare the indices of neighbouring areas or combined to produce annual difference maps. The former provides important information that may be directly relevant for farmers to improve the plants’ health during the year and to monitor the impact of different weather patterns, while the latter may be an indicator of long-term developments (such as depleted soil).

## Discussion

In the following, we discuss the benefits, implications and applications of our end-to-end framework and its product. We conclude with the potential of our map to increase sustainability along the cocoa supply chain.

Compared with existing mapping efforts, our framework promises a number of advantages. First, utilizing model ensembles in combination with aggregating over multiple satellite images of the same location allows for a confidence map in contrast to binary predictions. Consequently, an end user gains an additional degree of freedom when using our map. Depending on the concrete tasks, one can adapt the threshold of classifying cocoa according to one’s needs—for example, to fine-tune on a local region. Furthermore, the confidence map serves as guidance for measurements and improving predictions. While scores in the lower and upper ranges can be used to accurately and confidently take plant-specific measurements by decreasing the bias of false positives, uncertain predictions can be preferably checked on the ground to improve the model performance. Hence, non-profit organizations, initiatives and governments can drastically reduce human resources for on-the-ground surveys and mapping efforts. Mapping and protection efforts can be concentrated around and within protected areas. In case of reduced manpower, inspections on the ground can be focused on highly certain predictions. In addition to short-term forest clearance, it is possible to correlate long-term primary forest loss with cocoa encroachment in a highly accurate manner. We also tuned the confidence threshold on country-wide validation data. As demonstrated in the previous section, this results in a highly accurate binary cocoa map that can be used in various downstream tasks—for example, to specifically mask out vegetation areas not used for cocoa production to compute cocoa-specific vegetation indices such as the NDVI at the local community level, thanks to the map’s high resolution of 10 m.

A major finding of our mapping efforts is the substantial difference between the official harvested area in Ghana (1.63 Mha, average 2017–2020) and our total planted area estimation (2.7 Mha). Various reasons can partially explain this difference (which seems too substantial to be due only to young cocoa plantations detected by our map but not yet productive, and thus not accounted for in the harvested area). First, FAOSTAT numbers are based on imputed data, which are not as trustworthy for permanent crops due to the unreliability of figures reported by the corresponding country, particularly for cocoa and coffee^44^. In addition, it is known that up to 100,000 tonnes of cocoa beans per year have been smuggled back and forth across the border between the two countries^45,46^, resulting in skewed official production figures. Our map also differs from the official Ghanaian maps^26^, which are only partially mapping cocoa in the country. In particular, the Volta region is ignored completely, even though official production numbers (as seen in the ‘Production’ section) suggest that cocoa farming takes place in the eastern part of the country. Our mapping efforts predicted a total of 60,000 ha of additional cocoa plantations in the Volta region.

Our map demonstrates the massive role of cocoa in forest clearance in protected areas. Cocoa-driven deforestation is rooted in many interrelated factors, extensively explored in other studies^5,13,38^. At a basic level, farmers pursue cocoa in protected areas to meet essential livelihood needs (including income and food production) that have been disrupted due to declining productivity, civil unrest^13^, migration and population pressure in existing farming areas. In both countries, nearly all cocoa smallholders live under the poverty line, with daily wages of less than one dollar^6^. Average cocoa yields are low, mainly due to depleted soils, ageing and diseased trees, and low input use^47^. Concomitant to securing land rights, clearing natural forests to establish new cocoa farms provides farmers with temporarily fertile land and thus higher yields and income in the short run than using already cultivated sites^9^. Our findings stress the drastic need for fairer prices and improved government and company policies to support cocoa farmers’ adoption of improved practices. This must happen alongside stronger law enforcement to avoid rebound effects and preserve the remaining forests of Côte d’Ivoire and Ghana.

We have developed an end-to-end trainable framework to map cocoa in the world’s largest cocoa-producing countries, promising high accuracy and flexibility. Nevertheless, a few limitations have to be considered. We have demonstrated the applicability and usefulness of deep learning for automated crop identification with optical satellite imagery. Yet, the final map relies on multiple image acquisitions for each location to cope with atmospheric disturbances, as optical sensors are limited by cloud cover. Due to this limitation, while the map itself can be used to detect cocoa within protected areas, it is not yet possible to capture new cocoa plantations on a weekly or monthly update. Integrating radar-based observations (in particular synthetic aperture radar) as an additional input for our framework could probably reduce the number of images needed per location, ultimately increasing the update rate of our map. Combining historical satellite data with our map to detect past and current cocoa expansion rates is also an interesting future application. The proposed approach is a generic framework not limited to a specific region and is expected to generalize to new areas. Given reference data from new regions of interest, the model may be fine-tuned to adapt to local conditions and patterns. In particular, regions with similar landscape characteristics (for example, Cameroon or Nigeria) should only need small additional datasets, whereas adapting to countries such as Malaysia, Indonesia or Honduras with challenging growing practices (for example, high shade tree density or mixed cultivation) will probably require a lot more reference data.

We believe that our study vindicates automatic analysis of satellite imagery as a tool for large-scale mapping of cocoa and thereby presents a step forward in analysing the cocoa supply chain and its sustainability implications. Beyond the cocoa supply chain, this study also highlights the potential of using satellite imagery to derive the spatial extent of agricultural production in contexts with limited land documentation and therefore opens up opportunities to inform the design and implementation of public and private sustainability initiatives.

## Methods

### Data

As model input, we used publicly available optical satellite imagery to train and apply our predictor. Data for both countries were acquired from the Copernicus Sentinel-2 mission. Sentinel-2 images consist of 13 spectral bands, ranging from short-wave infrared to visible at a resolution of at most 10 m. We discarded bands with a resolution of 60 m and bilinearly upsampled all 20 m spectral bands to 10 m resolution, for a total of nine channels that served as input to our neural network. To make the model more robust towards atmospheric noise, cloudy recordings and sensor noise, we obtained in each Sentinel-2 grid tile the ten images with the lowest cloud cover within each six-month period, over a total observation window of three years between October 2018 and December 2021. Consequently, for each pixel location, we have up to 60 valid observations. In most cases, the number is smaller due to local cloud cover in some of the images (according to the Sentinel-2 basic cloud mask). During both training and testing, such cloudy samples were masked with ‘nodata’ values in a post-processing step.

We obtained ground truth from different data providers, mainly industrial partners, cocoa foundations and non-profit organizations. In total, we collected over 100,000 GPS-tracked cocoa farms and manually labelled over 10,000 background polygons of different sizes across both Ghana and Côte d’Ivoire. These negative samples, needed for supervised end-to-end learning, consist of villages, cities, rivers, lakes, open land, shrub land, forest and different commodities such as oil palm, rice and rubber. While cocoa has the largest number of individual polygons, the background samples have more than four times larger surface area due to the small size of cocoa farms (see the ‘Deep learning framework’ section on how we accounted for this class imbalance during training). Instead of splitting our dataset into training and validation parts at the farm level, we randomly cropped out large connected regions as validation areas to avoid biases caused by spatial correlation between nearby farms^48^. In total, we held out ~20% as validation data. For training, we projected and rasterized every ground-truth polygon to the corresponding Sentinel-2 tile, randomly chose a patch of 320 m × 320 m (32 pixels × 32 pixels) in which at least 10% of the pixels were labelled and extracted the corresponding patch from a randomly selected Sentinel-2 image. That procedure was repeated to generate hundreds of millions of input samples. The statistical strength afforded by this massive amount of training data is one reason for the good performance of our framework.

### In situ data

Independent of the training and validation data, we created a unique evaluation protocol by gathering additional test data on the ground. Together with our partners from industry, we designed a verification campaign by choosing over 2,000 random locations around ten different cities in Côte d’Ivoire, in such a way that they overlap neither with the training nor with the validation set. Each location was defined by a centre coordinate and an area around that centre point, which may vary in size and shape. Several teams were sent out to visit these predefined sites. Whenever possible, they were instructed to walk around the boundary of the area and to report back the estimated percentage of cocoa grown on the site. Furthermore, they were asked to note down any other commodity grown within the area—that is, an exemplary feedback would be that the area was occupied by “60% cocoa, 20% natural forest, 10% manioc, 10% palm trees”. If the majority (>50% of the total area) was cocoa, the location was considered as a positive cocoa sample, otherwise a negative (non-cocoa) one. The on-site verification lasted for more than six months, beginning in March 2021. In that dataset, the actual cocoa plantings are not geolocated within the polygons (smallholders in general grow multiple crops on their territory); thus, we evaluated our map on the farm level and counted a polygon as cocoa if a majority of its pixels fell into that class.

### Deep learning framework

With the aforementioned database, we were able to train end-to-end from raw spectral values, bypassing the manual design of predictive features. Instead, feature extraction was learned automatically. To that end, we employed a fully convolutional neural network based on the architecture proposed by Lang et al.^15^. The entry block of our model receives an image patch with nine Sentinel-2 bands and passes it through three consecutive residual blocks^49^ with learnable 1 × 1 convolutional filters. The output of that purely spectral, per-pixel analysis is then fed into a series of six residual blocks with 3 × 3 depth-wise separable convolutional layers^50^, which enable the network to exploit textural features (that is, spatial correlations between pixels). Next, the (normalized) vegetation height map is included by simply adding it to every channel of the intermediate feature map, and the result is fed through two further separable residual blocks to obtain the final feature representation. From it, the final output is computed with a single convolutional layer with two 1 × 1 filters, whose two-channel output is passed through a sigmoid transformation. This yields, at every 10 m × 10 m pixel, two positive output values that sum to 1 and can be interpreted as the probabilities for the presence and absence of cocoa. Since there are no downsampling operations and padding is applied in all residual blocks, the input resolution is retained, and one can directly compare the output to the ground-truth map. Additionally, as the network architecture is fully convolutional, it is not fixed to a specific spatial input size and can process image tiles of any size (subject to computing memory) during inference, reducing computation time during deployment.

We optimized the neural network’s weights by minimizing the Dice coefficient, also called the overlap index. The Dice coefficient loss is defined as

3 $${{{\mathcal{L}}}}=\mathop{\sum}\limits_{c}\left(1-\frac{2\mathop{\sum}\limits_{i}{p}_{ci}{g}_{ci}+\epsilon }{\mathop{\sum}\limits_{i}{p}_{ci}+\mathop{\sum}\limits_{i}{g}_{ci}+\epsilon }\right),$$

where *c* is the number of classes, *i* the pixel index, and *p* and *g* are the prediction and ground truth, respectively. For numerical stability, a small *ϵ* is added to the numerator and denominator. The Dice loss is a common loss function used in medical image segmentation, as it is more robust under data imbalance than loss functions based on standard cross-entropy ^51,52^. As our training data are sparse within patches (a training patch only needs to have ground truth at >10% of its pixels), we further masked out all pixels without a ground-truth label and computed the Dice loss selectively only for the labelled part. Patches are combined into batches of size 32. The network is trained for 500 epochs, with each epoch consisting of 40,000 iterations, using the Adam optimizer^53^ with a base learning rate of 10^−5^. On our high-performance computing infrastructure, one training run took slightly more than five days.

Confidence (and uncertainty) estimates from individual deep neural networks are known to be poorly calibrated^54^. For better uncertainty calibration, we employed a model ensemble^21^. Ten replicas of the neural network just described were trained independently on the same dataset, with different random initializations and different (random) batches. Additionally, averaging estimates over multiple observations diminishes the influence of faulty classifications due to noisy observations. Model ensembling further allowed us to compute CIs on different estimators such as area. We thresholded each of the continuous maps generated by the ten independent models and computed an area estimate per model. We obtained CIs assuming an underlying *t*-distribution with nine degrees of freedom as follows:

4 $$\left[\mu -{t}_{n-1}(c)\times \frac{\sigma }{\sqrt{n}},\mu +{t}_{n-1}(c)\times \frac{\sigma }{\sqrt{n}}\right],$$

where *μ* is the sample mean, *σ* is the sample standard deviation, *c* is the confidence level and *t*_*n*−1_ is the critical value with (*n* − 1) degrees of freedom.

### Vegetation height map

Besides the nine Sentinel-2 optical bands, our network ingests a dense vegetation height map as an auxiliary input channel (see the previous section). The per-pixel vegetation heights were derived from Sentinel-2 optical images with a deep learning method originally developed and tested for Southeast Asia ^55^. That method also employs a fully convolutional neural network, but one that is trained to regress canopy height from Sentinel-2 imagery, using as the training target sparse canopy height samples extracted from the GEDI mission of the National Aeronautics and Space Administration^56^. Due to the sparsity of GEDI’s LiDAR footprints, we trained this model not only on samples from Côte d’Ivoire and Ghana but also on an extended training area covering all of West Africa. Despite being trained on sparse data, the model outputs a dense canopy height map with 10 m ground sampling distance. Although the vegetation height map is also derived from Sentinel-2 and therefore is arguably just another feature that could be learned from the input imagery, there are two reasons to directly incorporate it as an input channel. First, cocoa trees are known to grow to a maximum height of ~8 m (refs. ^57,58^) (sometimes under higher shade trees, but these scattered trees protruding from the lower cocoa plants also provide a distinctive height pattern). Vegetation height is thus an obvious predictive feature simply for its ability to identify high vegetation as not being cocoa. It therefore seems reasonable to simplify the learning process and save model capacity by supplying it directly. The second (and more essential) reason why we expect the separate tree height estimator to improve the estimates is that it brings in additional information. While the vegetation height map is indeed based on the same input, it has not been learned from the same output. Rather, the cocoa mapping pipeline benefits from the additional, strong supervision signal of the GEDI LiDAR measurements, which is baked into the canopy height map.

### Reporting summary

Further information on research design is available in the Nature Portfolio Reporting Summary linked to this article.

## Supplementary information

Supplementary Information Supplementary Figs. 1 and 2 and Tables 1 and 2.

Reporting Summary

## Data Availability

The cocoa probability map and its thresholded version will be released for download and will be available in the Google Earth Engine. Both maps can be explored interactively in the following Google Earth Engine application: https://nk.users.earthengine.app/view/cocoa-map.

## Code availability

The code is available at https://github.com/D1noFuzi/cocoamapping/.
