# Supplementary material for: Cocoa plantations are associated with deforestation in Côte d’Ivoire and Ghana
Source: Nat Food. 2023 May 22;4(5):384–93. doi: 10.1038/s43016-023-00751-8 (PMC10208960; doi:10.1038/s43016-023-00751-8)
Supplement: Supplementary file 1 — Supplementary Figs. 1 and 2 and Tables 1 and 2. [file 43016_2023_751_MOESM1_ESM.pdf]

nature food

Article https://doi.org/10.1038/s43016-023-00751-8

Cocoa plantations are associated with deforestation in Côte d'Ivoire and Ghana

In the format provided by the authors and unedited

Country	Region	Derived planted area (Mha)	Land cover (%)
Ghana	Western region	1.09	44.6
	Eastern region	0.29	15.5
	Central region	0.32	33.0
	Volta region	0.06	2.7
	Brong-Ahafo region	0.29	7.3
	Ashanti region	0.66	26.7
Côte d'Ivoire	Yamoussoukro region	0.01	6.2
	Woroba region	0.03	1.0
	Montagnes region	0.95	31.2
	Sassandra-Marahoué region	0.54	22.3
	Lacs region	0.16	6.0
	Lagunes region	0.47	23.8
	Gôh-Djiboua region	0.60	38.5
	Bas-Sassandra region	1.18	42.9
	Comoé region	0.36	24.7
	Zanzan region	0.03	0.9

Suppl. Table 1: **Total planted area and relative land cover per region in Côte d'Ivoire and Ghana.** Regions are defined following official administrative boundaries.

Côte d'Ivoire			Ghana		
Protected area	Cocoa (ha)	Land cover (%)	Protected area	Cocoa (ha)	Land cover (%)
Taï National. (NP)	1,552	0.3	Subri River (FR)	726	1.2
Rapide Grah (CF)	96,089	60.1	Bia Tawya (FR)	41,727	77.1
Haute Dodo (CF)	75,281	51.3	Tain Tributa. (FR)	132	0.2
Scio (CF)	90,418	68.2	Krokosua Hil. (FR)	5,099	10.8
Niegre (CF)	108,256	81.8	Anlo-Keta la.	0	0.0
Seguela (CF)	25,564	22.3	Tano Ofin (FR)	858	2.1
Mt. Sassandr. (CF)	54,946	49.0	Ankasa (RR)	776	2.1
CFNU No.72 (CF)	51,080	48.7	Kyabobo Nati. (NP)	6	0.0
Marahoue Nat. (NP)	8,352	8.1	Kogyae (SNR)	0	0.0
Mont Sangbe . (NP)	0	0.0	Bia North (FR)	1,010	2.8
Songan/Tamin (CF)	35,763	39.0	Subuma (FR)	997	2.8
Mabi/Yaya (CF)	16,299	18.2	Sui River (FR)	3,497	9.8
CFNU No.77 (CF)	8,730	9.8	Mpameso (FR)	741	2.2
CFNU No.58 (CF)	62,359	72.3	Bia National. (NP)	891	2.8
Go Bodienou (CF)	39,275	47.9	Bia Resource. (RR)	723	2.3
Yarani (CF)	2,720	3.7	Boin River (FR)	764	2.5
Issia (CF)	18,603	27.9	Bia National. (UBR)	18	0.1
Nibi Hana (CF)	29,982	48.3	Manzan (FR)	15,512	56.1
Duekoue (CF)	18,096	29.7	Asenanyo (FR)	2,232	8.6
Sangoue (CF)	21,373	36.1	Asukese (FR)	87	0.3
Bayota (CF)	18,204	31.5	Yoyo (FR)	270	1.2
Mt. Ko (CF)	5,295	9.2	Bomfoun (FR)	0	0.0
Dogodou (CF)	21,308	39.9	Subin (FR)	533	2.3
Irobo (CF)	13,225	25.3	Draw River (FR)	193	0.9
De (CF)	16,386	31.5	Atewa Range (FR)	210	1.0
Tene (CF)	6,763	14.5	Tano Ehuro (FR)	16,275	77.6
Besso (CF)	19,732	44.2	Kakum (NP)	256	1.2
Beki Bosse M. (CF)	12,096	28.8	Tano Nimri (FR)	669	3.3
Diambarakrou (CF)	26,581	64.2	Pamu Berekum (FR)	89	0.5
CFNU No.43 (CF)	12,993	32.1	Bia Tano (FR)	195	1.0
CFNU No.66 (CF)	1,141	3.0	Afram Headwa. (FR)	60	0.3
Plaine des E. (CF)	7,489	20.2	Bodi (FR)	12,947	70.0
Kamesso (CF)	10	0.0	Chai River (FR)	22	0.1
Abeanou (CF)	4,550	13.6	Bandai Hills. (FR)	0	0.0
Tanoe (CF)	18,332	55.7	Fure Headwat. (FR)	669	4.0
Monogaga (CF)	20,937	65.3	Oda River (FR)	306	1.8
Gouin (CF)	19,099	61.2	Bonsa Ben (FR)	178	1.1
Mont Peko Na. (NP)	6,479	21.5	Bonsa River (FR)	1,059	6.5
Seguie (CF)	8,850	29.9	Worobong Nor. (FR)	1,852	11.3
CFNU No.50 (CF)	2,862	9.7	Dadieso (FR)	303	1.9
CFNU No.30 (CF)	3,552	12.2	Fure River (FR)	161	1.0
Yalo (CF)	2,941	10.1	Assin Attand. (RR)	387	2.5
CFNU No.40 (CF)	2,760	9.8	Desiri (FR)	9,383	60.2
N'Zo Fauna R. (PFR)	13	0.0	Boabeng-Fiem.(WS)	0	0.0
Mopri (CF)	10,742	39.6	Togo Plateau (FR)	301	2.0
Arrah (CF)	7,809	29.6	Tano Anwia (FR)	212	1.4
CFNU No.56 (CF)	12,007	47.2	Tonton (FR)	161	1.1
CFNU No.31 (CF)	228	0.9	Bosomkese (FR)	250	1.7
Tiapleu (CF)	5,416	22.0	Sukusuki (FR)	12,053	83.6
Mt. Tia (CF)	14,886	62.0	Southern Sca. (FR)	1,486	10.7
Ebrinenou (CF)	12,744	53.6	Upper Wassaw (FR)	3,198	23.6
Moyenne Mara. (CF)	1,280	5.7	Bonsam Bepo (FR)	807	6.1
Azagny Natio. (NP)	249	1.1	Nini-Suhien (NP)	665	5.1

Côte d'Ivoire			Ghana		
Protected area	Cocoa (ha)	Land cover (%)	Protected area	Cocoa (ha)	Land cover (%)
CFNU No.53 (CF)	2,619	12.5	Awura (FR)	0	0.0
Agbo (CF)	12,029	57.8	Neung South (FR)	399	3.1
Zuoke (CF)	10,825	52.1	Pra Anum (FR)	984	7.6
Abokouamekro. (NR)	4	0.0	Opon Mansi (FR)	646	5.0
Niouniourou (CF)	12,375	64.5	Opro River (FR)	19	0.2
Hein (CF)	2,546	13.9	Bosomoa (FR)	0	0.0
Tyemba (CF)	0	0.0	Esukawkaw (FR)	124	1.0
Zagoreta (CF)	8,844	50.0	Southern Sca. (FR)	277	2.3
Vavoua (CF)	4,186	23.8	Anhwiaso Eas. (FR)	1,033	8.5
Brassue (CF)	5,034	28.8	Boi Tano (FR)	210	1.7
Semien Flans. (CF)	4,536	26.0	Tinte Bepo (FR)	316	2.6
Elroukro (CF)	2,661	15.3	Ayum (FR)	134	1.1
Adzope (CF)	6,500	38.3	Worobong Sou. (FR)	104	0.9
Tos (CF)	1,477	8.9	Bowiye Range (FR)	222	2.0
Kavi (CF)	9,125	57.8	Chirimfa (FR)	3	0.0
Oume Doka (CF)	4,213	27.0	Bimpong (FR)	233	2.2
Marahoue (CF)	2,789	18.0	Pra Suhyien . (FR)	373	3.6
Tankesse (CF)	2,307	15.3	Bura River (FR)	113	1.1
Kravassou (CF)	5,468	37.2	Nkrabia (FR)	244	2.4
Ira (CF)	5,871	41.1	Tano Suhyien (FR)	407	4.5
Manzan (CF)	5,596	39.9	Mankrang (FR)	1	0.0
Mt. De (CF)	1,900	14.0	Kwamisa (FR)	234	2.8
Bouafle (CF)	2,163	16.1	Fum Headwate. (FR)	158	1.9
Dassieko (CF)	4,692	34.8	Kabo River (FR)	1,544	19.0
CFNU No.39 (CF)	7,484	55.9	Dampia Range (FR)	414	5.1
Kassa (CF)	2,617	19.9	Dome River (FR)	533	6.7
Offumpo (CF)	6,637	51.2	Pra Suhyien (FR)	76	1.0
Ananguie (CF)	7,712	60.5	Asuokoko Riv. (FR)	157	2.0
Nizoro (CF)	7,477	59.4	Afrensu Broh. (FR)	0	0.0
Mt. Sainte/C. (CF)	7,612	62.7	Tano Suraw E. (FR)	2,216	28.6
Bableu (CF)	5,249	43.6	Kalakpa (RR)	0	0.0
Krozalie (CF)	5,486	48.7	Asubima (FR)	1	0.0
Mt. Bolo (CF)	4,929	44.0	Bosumtwi Ran. (FR)	1,089	14.1
CFNU No.68 (CF)	6,683	59.7	Bomfobiri (WS)	333	4.3
Mando (CF)	2,016	18.7	North Bandai. (FR)	0	0.0
CFNU No.42 (CF)	4,387	41.7	Bonkoni (FR)	53	0.7
CFNU No.34 (CF)	4,946	47.2	Jema Asemkro. (FR)	1,925	27.1
Kouadikro (CF)	246	2.4	Ndumfri (FR)	156	2.2
CFNU No.36 (CF)	96	0.9	Apepesu Rive. (FR)	1,084	16.2
Bolo (CF)	4,676	47.1	Muro (FR)	183	2.8
CFNU No.74 (CF)	1	0.0	Sawsaw (FR)	9	0.1
CFNU No.33 (CF)	2,514	27.3	Mirasa Hills (FR)	191	3.0
Goudi (CF)	4,548	50.2	Totua Shelte. (FR)	65	1.0
CFNU No.57 (CF)	589	6.5	Northern Sca. (FR)	0	0.0
CFNU No.38 (CF)	2,684	30.7	Nsuensa (FR)	10	0.2

Suppl. Table 2: **Extended table of the one hundred protected areas in Côte d'Ivoire and Ghana.** Entries are ordered according to their total protected area (within suitable cocoa growing area). CFNU = classified forest unknown name, CF = classified forest, NP = national park, WS = wildlife sanctuary, FR = forest reserve, RR = resource reserve.

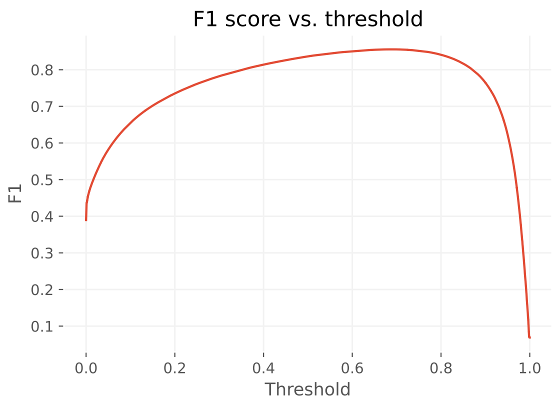

(a)

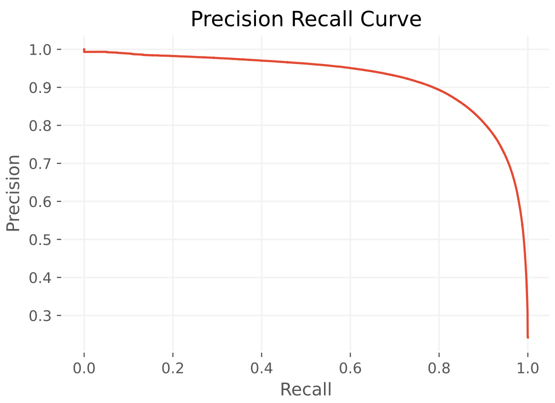

(b)

Suppl. Figure 1: **Threshold optimization.** (a) F1 score of cocoa pixels from the validation set in relation to the threshold. (b) Precision recall curve of all cocoa pixels from the validation set.

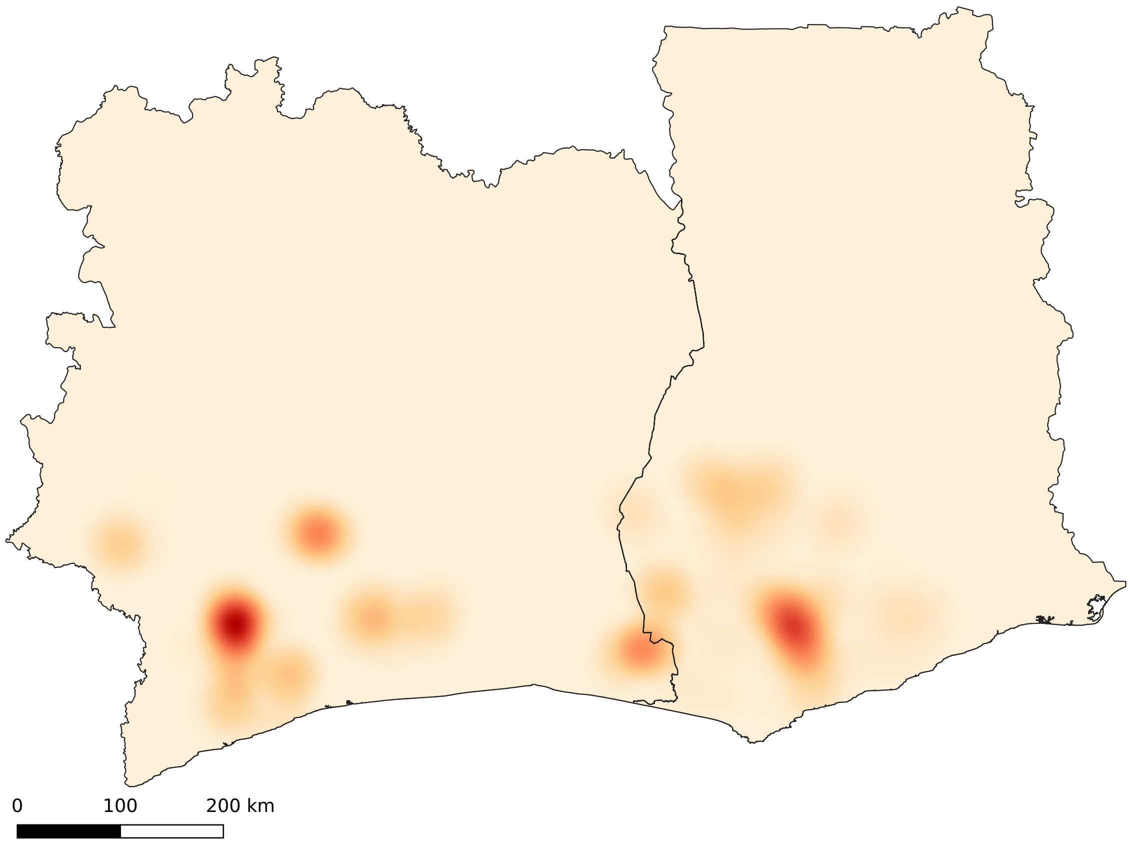

Suppl. Figure 2: **Spatial distribution of our test set.** Heatmap depicts approximate geolocation of in situ test set in Côte d'Ivoire and accompanying test set in Ghana.
