# Supplementary material for: Cocoa plantations are associated with deforestation in Côte d’Ivoire and Ghana
Source: Nat Food. 2023 May 22;4(5):384–93. doi: 10.1038/s43016-023-00751-8 (PMC10208960; doi:10.1038/s43016-023-00751-8)
Supplement: Supplementary file 2 — Reporting Summary [file 43016_2023_751_MOESM2_ESM.pdf]

nature portfolio

Corresponding author(s): Nikolai Kalischek

Last updated by author(s): Apr 3, 2023

Reporting Summary

Nature Portfolio wishes to improve the reproducibility of the work that we publish. This form provides structure for consistency and transparency in reporting. For further information on Nature Portfolio policies, see our Editorial Policies and the Editorial Policy Checklist.

Statistics

For all statistical analyses, confirm that the following items are present in the figure legend, table legend, main text, or Methods section.

n/a	Confirmed	
☐	☒	The exact sample size (*n*) for each experimental group/condition, given as a discrete number and unit of measurement
☐	☒	A statement on whether measurements were taken from distinct samples or whether the same sample was measured repeatedly
☒	☐	The statistical test(s) used AND whether they are one- or two-sided<br>*Only common tests should be described solely by name; describe more complex techniques in the Methods section.*
☐	☒	A description of all covariates tested
☐	☒	A description of any assumptions or corrections, such as tests of normality and adjustment for multiple comparisons
☐	☒	A full description of the statistical parameters including central tendency (e.g. means) or other basic estimates (e.g. regression coefficient) AND variation (e.g. standard deviation) or associated estimates of uncertainty (e.g. confidence intervals)
☒	☐	For null hypothesis testing, the test statistic (e.g. *F*, *t*, *r*) with confidence intervals, effect sizes, degrees of freedom and *P* value noted<br>*Give P values as exact values whenever suitable.*
☒	☐	For Bayesian analysis, information on the choice of priors and Markov chain Monte Carlo settings
☒	☐	For hierarchical and complex designs, identification of the appropriate level for tests and full reporting of outcomes
☒	☐	Estimates of effect sizes (e.g. Cohen's *d*, Pearson's *r*), indicating how they were calculated

Our web collection on statistics for biologists contains articles on many of the points above.

Software and code

Policy information about availability of computer code

Data collection	No software was used to collect data.
Data analysis	The data analysis, i.e. model training and evaluation was fully developed in Python3.0 with no additional commercial software. All code can be found at https://github.com/D1noFuzi/cocoamapping/.

For manuscripts utilizing custom algorithms or software that are central to the research but not yet described in published literature, software must be made available to editors and reviewers. We strongly encourage code deposition in a community repository (e.g. GitHub). See the Nature Portfolio guidelines for submitting code & software for further information.

Data

Policy information about availability of data

All manuscripts must include a data availability statement. This statement should provide the following information, where applicable:

- Accession codes, unique identifiers, or web links for publicly available datasets
- A description of any restrictions on data availability
- For clinical datasets or third party data, please ensure that the statement adheres to our policy

Data collection is two-fold for our submission. All input data, including all Sentinel-2 images and vegetation height maps for Cote d'lvoire and Ghana are publically downloadable from data providers such as Copernicus Open Access Hub and Google Earth Engine in case of the vegetation height map.

The ground truth data of cocoa farms and additional background polygons are gathered from different commercial data providers and cannot be shared publically.

The cocoa probability map and its thresholded version are released for download and available in the Google Earth Engine. Both maps can be explored interactively in the following Google Earth Engine application:
https://nk.users.ea rthengine.app/view/cocoa-map.

Human research participants

Policy information about studies involving human research participants and Sex and Gender in Research.

Reporting on sex and gender	Not Applicable.
Population characteristics	Not Applicable.
Recruitment	Not Applicable.
Ethics oversight	Not Applicable.

Note that full information on the approval of the study protocol must also be provided in the manuscript.

Field-specific reporting

Please select the one below that is the best fit for your research. If you are not sure, read the appropriate sections before making your selection.

☐ Life sciences ☐ Behavioural & social sciences ☒ Ecological, evolutionary & environmental sciences

For a reference copy of the document with all sections, see nature.com/documents/nr-reporting-summary-flat.pdf

Ecological, evolutionary & environmental sciences study design

All studies must disclose on these points even when the disclosure is negative.

Study description	We investigate crop classification based on high-resolution satellite imagery utilizing deep neural networks. In particular, we combine cocoa plantation data with publicly available satellite imagery in a deep learning framework and create high-resolution, large-scale maps of cocoa plantations for the two largest producers of cocoa, Côte d'lvoire and Ghana.
Research sample	We use a large dataset of geo-referenced cocoa farms including over 100,000 samples and over 10,000 samples of non-cocoa sites. The enormous amount of data is needed to train a deep neural network to correctly map cocoa at large scale.
Sampling strategy	We did not use any specific sample size calculation, as it is generally known that deep learning needs vast amounts of data tobe properly trained. Additionally, the bigger the dataset, the better the overall performance. Hence, we tried to collect as many samples as possible.
Data collection	The cocoa data was collected from 2015 onwards by several commercial data providers. As cocoa plants are generally productive for many years, farms are unlikely to have changed or degraded over the past years. Additionally, we trained the network on satellite imagery from 2018 to 2021 to enforce a higher overlap.
Timing and spatial scale	As mentioned above, data was collected from 2015 onwards with yearly updates ofthe database. Spatially, data was naturally collected in the main cocoa growing regions.
Data exclusions	We excluded all obvious labelling mistakes such as polygons located within villages but yet classified as cocoa farms.
Reproducibility	We naturally included reproducability in the experiments by training multiple models on the same data, i.e. ten models and training runs in total.
Randomization	We split our dataset into two groups, training and validation. We randomly crop out large connected regions as validation areas, so as to avoid biases caused by spatial correlation between nearby farms.<br>Additionally, we test our model on a in situ test set, that was gathered on ground, including over 2000 random locations.
Blinding	We used blinding for our in situ test set, i.e. we collected 2000 random locations and sent out teams on ground to classify the location without knowing what our model predicted for that specific location.

Did the study involve field work? ☒ Yes ☐ No

Field work, collection and transport

Field conditions	Field work was conducted by local teams in Cöte d'lvoire to acquire an independent test set.
Location	Côte d'Ivoire
Access & import/export	Data was collected within agricultural areas, hence no additional permits were needed.
Disturbance	No disturbances during data collection.

Reporting for specific materials, systems and methods

We require information from authors about some types of materials, experimental systems and methods used in many studies. Here, indicate whether each material, system or method listed is relevant to your study. If you are not sure if a list item applies to your research, read the appropriate section before selecting a response.

Materials & experimental systems

n/a	Involved in the study
☒	☐ Antibodies
☒	☐ Eukaryotic cell lines
☒	☐ Palaeontology and archaeology
☒	☐ Animals and other organisms
☒	☐ Clinical data
☒	☐ Dual use research of concern

Methods

n/a	Involved in the study
☒	☐ ChIP-seq
☒	☐ Flow cytometry
☒	☐ MRI-based neuroimaging
